# Supplementary material for: Regular rhythmic and audio-visual stimulations enhance procedural learning of a perceptual-motor sequence in healthy adults: A pilot study
Source: PLoS One. 2021 Nov 15;16(11):e0259081. doi: 10.1371/journal.pone.0259081 (PMC8592429; doi:10.1371/journal.pone.0259081)
Supplement: S1 Table — Table for the interpretation of each Bayes factors level. Adapted from Jeffreys (1998). (DOCX) [file pone.0259081.s001.docx]

| BF10 | Support for hypothesis |
| --- | --- |
| < 0.01 | Decisive evidence for H_0_ |
| 0.03-0.01 | Very strong evidence for H_0_ |
| 0.10-0.03 | Strong evidence for H_0_ |
| 0..33-0.10 | Substantial evidence for H_0_ |
| 0.33-1 | Anecdotal evidence for H_0_ |
| 1 | No evidence |
| 1-3 | Anecdotal evidence for H_1_ |
| 3-10 | Substantial evidence for H_1_ |
| 10-30 | Strong evidence for H_1_ |
| 30-100 | Very strong evidence for H_1_ |
| >100 | Decisive evidence for H_1_ |

Table for the interpretation of each Bayes factors level. Adapted from Jeffreys (1998).
