# Supplementary material for: Regular rhythmic and audio-visual stimulations enhance procedural learning of a perceptual-motor sequence in healthy adults: A pilot study
Source: PLoS One. 2021 Nov 15;16(11):e0259081. doi: 10.1371/journal.pone.0259081 (PMC8592429; doi:10.1371/journal.pone.0259081)
Supplement: Attachment — Submitted filename: Responses to reviewers.docx [file pone.0259081.s005.docx]

Dear Professor Fetsch and reviewers,

We would like to thank you for reviewing our manuscript entitled “*Rhythmic and audio-visual stimulations enhance procedural learning of a perceptual-motor sequence in healthy adults: a pilot study*” for publication in PLOS ONE. All of you did very insightful comments and proposed relevant changes that highly improved the quality of the manuscript.

The manuscript has been revised for better readability according to the suggestions. We responded to all of the remarks with associated changes highlighted in blue.

In accordance with PLOS ONE’s policy, we uploaded anonymized data set as Supporting Information files and we adjusted titles and Figures’ style to meet PLOS ONE’s requirements.

All authors have reviewed and agreed to the submission of the revised manuscript. We hope that our responses fix all the issues that you raised and that the manuscript is now acceptable for publication. Please do not hesitate to contact me if there are any questions.

**Editor:** In addition to the scientific concerns, I would like to point out that the Data Availability requirement has not been met for this submission. Specifically, the data availability questions state: "Stating ‘data available on request from the author’ is not sufficient. If your data are only available upon request, select ‘No’ for the first question [Data Availability] and explain your exceptional situation in the text box." In most cases, PLOS ONE policy requires data to be deposited in a public repository or submitted as supplementary information.

1. Please ensure that your manuscript meets PLOS ONE's style requirements, including those for file naming. The PLOS ONE style templates can be found at

https://journals.plos.org/plosone/s/file?id=wjVg/PLOSOne_formatting_sample_main_body.pdf and <https://journals.plos.org/plosone/s/file?id=ba62/PLOSOne_formatting_sample_title_authors_affiliations.pdf>

- All the titles’ style has been changed to meet PLOS ONE’s requirements. Figures have been renamed and caption placed under.

2. Please ensure that you have provided sufficient detail on participant recruitment in the Methods section.

- We agree that participants recruitment was not clear enough. The sentence following sentence was added “Participants were undergraduate students pursuing sports science courses in Toulouse university.” (p.5)

3. Thank you for including your ethics statement:  "All participants provided informed consent prior to data collection. Procedure was in accordance with the Declaration of Helsinki and followed institutional ethics board guidelines for research on humans (IRB00003888)".

- We included our ethics statements in the corresponding section.

3.1. Please amend your current ethics statement to include the full name of the ethics committee/institutional review board(s) that approved your specific study.

3.2. Please amend your current ethics statement to confirm that your named institutional review board or ethics committee specifically approved this study.

- We completed our ethics statements with the sentence “The study was conducted in accordance with the Declaration of Helsinki and approved by the Inserm (Institut National de la Santé et de la Recherche Médicale) ethical committee (Institutional Review Board IRB00003888 - agreement n°14–156).” (p.5-6).

4.3. Please provide additional details regarding participant consent. In the ethics statement in the Methods and online submission information, please ensure that you have specified what type you obtained (for instance, written or verbal, and if verbal, how it was documented and witnessed). If your study included minors, state whether you obtained consent from parents or guardians. If the need for consent was waived by the ethics committee, please include this information.

Once you have amended this/these statement(s) in the Methods section of the manuscript, please add the same text to the “Ethics Statement” field of the submission form (via “Edit Submission”).

For additional information about PLOS ONE ethical requirements for human subjects research, please refer to http://journals.plos.org/plosone/s/submission-guidelines#loc-human-subjects-research.

- We clarified how consent was obtained “Before the experiment beginning, all volunteers provided a verbal informed consent and documented a written form specifying their motivation to participate to the study.” (page 5-6).

 4.  We note that you have indicated that data from this study are available upon request. PLOS only allows data to be available upon request if there are legal or ethical restrictions on sharing data publicly. For more information on unacceptable data access restrictions, please see http://journals.plos.org/plosone/s/data-availability#loc-unacceptable-data-access-restrictions.

In your revised cover letter, please address the following prompts:

a) If there are ethical or legal restrictions on sharing a de-identified data set, please explain them in detail (e.g., data contain potentially sensitive information, data are owned by a third-party organization, etc.) and who has imposed them (e.g., an ethics committee). Please also provide contact information for a data access committee, ethics committee, or other institutional body to which data requests may be sent.

b) If there are no restrictions, please upload the minimal anonymized data set necessary to replicate your study findings as either Supporting Information files or to a stable, public repository and provide us with the relevant URLs, DOIs, or accession numbers. For a list of acceptable repositories, please see http://journals.plos.org/plosone/s/data-availability#loc-recommended-repositories.

We will update your Data Availability statement on your behalf to reflect the information you provide.

🡪 Thank you for this remark, we uploaded anonymized data set as Supporting Information files.

**Reviewer #1:** The authors investigate the effects of auditory stimulations on learning a visuomotor sequence (serial reaction time task). Their findings suggest that, when these stimulations are congruent with the task and/or rhythmic at a regular tempo, they promote procedural learning. Their findings are interesting, and indeed show that auditory stimulations affect procedural learning of a motor sequence. I have some comments/questions as to whether this effect amounts to enhancement; my reservations may be resolved with some additional clarifications.

- Thanks for your Reviewing. Before to respond to all of your comments, please note that Reviewer 2 asked to change the name of the condition. Accordingly, “VO”, “CAV”, “NCAV”, “RAS”, “IAS”, “QRAS” and “rhythmic auditory stimulation” are replaced by “VisOnly”, “CongrAV”, “NonCongrAV”, “RythAud”, IrregAud, FastRyth” and “Regular auditory stimulation” (RegAud). Modifications are made to the title, abstract and where appropriate throughout the manuscript in particular in the Material-Methods, Results, and Discussion sections.

1) The evidence for enhancement comes from studying specific learning (comparing performance in late learning of a sequence vs. subsequent performance in a random sequence block). The reaction times are similar across groups, but the error rates are different, which is the evidence for enhancement in the RAS and CAV groups.

While SRTT studies seem to mostly rely on RT analysis, I agree with the authors that the error rate is also important and an indication of learning. However, one issue with looking at error rates (and their differences) is that they might be more likely to have floor/ceiling effects – for example, a participant might have little in terms of errors in the random block (B6), thus can't be much better in B5. In particular, maybe the VO group (essentially the “vanilla” SRTT group, against which we compare for enhancement) is simply pretty good in both B5 and B6, with little errors.

So, given the importance of errors in justifying the paper’s thesis – that there is enhancement of learning – it will be helpful to see the absolute error rates during each block (like Figure 2, but for errors), rather than just the difference in error rates between B5 and B6.

- We agree with R1. The figure representing the absolute error rates during each block was added as Figure 4a.

2) What could obscure this enhancement when it comes to general learning? If we assume general learning encompasses specific learning (assessed by comparing B5 with B6) among other things, one would expect that increases in specific learning would increase general learning as well.

- Thanks for your remark. We clarified in the manuscript that specific learning and general learning are two distinct processes by adding a novel paragraph in the discussion section.

***“Specific learning can occur without general learning***

In two conditions (IrregAud and FastRhyth) we found specific learning without general learning. It is in accordance with previous findings showing that it is possible to enhance general learning but not sequence-specific learning (see 74). Thus, our results support the idea that general and specific learning are two distinct processes which are subserved by distinct neural correlates (74). The first one corresponds to stimulus-based mappings and the second one to internalized sequence representation, or response-based mappings (e.g., 75,76).”

- 74. Chan, R. W., Alday, P. M., Zou-Williams, L., Lushington, K., Schlesewsky, M., Bornkessel-Schlesewsky, I., & Immink, M. A. (2020). Focused-attention meditation increases cognitive control during motor sequence performance: Evidence from the N2 cortical evoked potential. *Behavioural brain research*, 384, 112536.
- 75. Tubau, E., Hommel, B., & López-Moliner, J. (2007). Modes of executive control in sequence learning: from stimulus-based to plan-based control. *Journal of Experimental Psychology: General*, 136(1), 43.
- 76. Abrahamse, E., Ruitenberg, M., De Kleine, E., & Verwey, W. B. (2013). Control of automated behavior: insights from the discrete sequence production task. *Frontiers in human neuroscience*, 7, 82.

3) Another limitation in comparing learning across the different groups is that they start from different baselines: e.g., there are large differences in reaction times in B1, before much learning is supposed to occur. In other words, there might be a confound due to differences in baseline performance.

- Thanks for this relevant comment. However, we have taken care to compare groups before the learning phases with B0 as a baseline, and there was no difference between conditions. Our results show that all groups started from the same baseline. Thus, we assume that the differences in the subsequent learning blocks are more due to the differential conditions than to differences in baseline. We also ensured that there was no significant correlation between the performance at the first block of practice (B0) and the SRTT learning score.

Clarifications about B0 have been added in the manuscript: “1 Block of familiarization (B0) displaying a sequence of 10 positions repeated 10 times (100 stimulations). It was performed in the same condition as the following blocks in order and aimed to make sure that there were no significant differences between the groups in performance at the beginning of the experiment (B0 can be considered as a baseline)” (p.8) and “Each sequence was attributed in a counterbalanced manner to the participants for each condition. The selected sequence of B0 was different from this selected for B1 to B5.” (p.8).

4) As a minor point – auditory stimuli might also act as cues to retrieve the previously learned sequence. So, during the random block, auditory stimuli might promote retrieval of the sequence (even though it is not helpful at that block) – in that way, we could see an over-expression of the previously learned sequence, which would not necessarily mean that the sequence was learned to a greater extent previously.

- This is an interesting idea. However, given that each position was not associated with a specific sound, it seems unlikely. We used a unique auditory stimulus (500Hz, 100ms sinewave) for all positions.

Moreover, we added in the manuscript (p. 9-10) that “In the conditions CongrAV, NonCongrAV, RhythAud, IrregAud and FastRhyth, the auditory stimuli were presented from Block 0 to Block 6. Presenting B0 without auditory stimuli might have benefit more the VisOnly condition than the other conditions. Moreover, a removal of the auditory stimuli in the random block (B6) would have induce a double change (sequence and auditory stimuli) for participants and it would have been difficult to determine if decrease change in performance would be due to a change in the sequence or the removal of the auditory stimuli.”.

5) Participants might be learning the sequence but also learning to deal with the auditory stimulation – it might be helpful to show that their performance was stable *before* the sequence was introduced.

- We agree with this comment, so we introduced the block B0 in the Figures 2a, 2b, 3a, 3b, 4a and 4b. We confirm that in this preliminary block there were no differences in RT or errors between conditions.

Errors and requests for clarification:

1) The caption in Figure 2 does not match the legend in terms of describing the different curves (*for the purpose of my review, I assumed the legend is correct*): it mentions RAS as yellow circles, whereas the legend shows them as purple squares; QRAS as gray circles, whereas the legend shows them as yellow triangles, etc. Please make sure all the colors/symbols mentioned in the caption are correct. The caption also mentions * / # that are not shown in the figure. Captions for figures 3a/3b also mention asterisks that are not shown.

- Thanks for this observation. The caption has been corrected to match the Figures. “Figure 2a: Reaction Times for general learning (from B1 to B5) and for specific learning (from B5 to B6) of all Conditions. and 2b: z-scores of Reaction Times for general learning (from B1 to B5) and for specific learning (from B5 to B6) of all Conditions. Regular Auditory Stimulations (RhythAud in purple squares), Congruent Audio-Visual (CongrAV in blue squares), Visual Only (VisOnly in green triangles), Non-Congruent Audio-Visual (NonCongrAV in red triangles), Irregular Auditory Stimulations (IrregAud in grey triangles) and Quick Rhythmic Auditory Stimulations (FastRhyth in yellow triangles).

2) p.15, second to last line: “a larger improvement of errors” – is it meant “a larger increase of errors”?

- Thanks for this correction. “improvement” was replaced by “increase” (p.15).

3) Clarification: Page 4 “… than with irregular metrics (i.e. isochronous intervals)” - Aren’t isochronous intervals also regular tempo?

- Thanks for this clarification. Here we wanted to say: “irregular intervals”. It was changed in the text (p.4).

4) Having “results” as a subtitle for the methods section (2.6) might be not helpful since this is also section 3

- Thanks for this comment. The subtitle 2.6 “results” was removed.

5) Typos on Page 16, bottom: one of these two variable*s* decrease*s*… the performance increase*s*…. The IAS condition did not led -> lead

- Thanks for these corrections. Both were modified in the text: “one of these two variables decreases” and “The IAS condition did not lead” (p.16).

**Reviewer #2**: The present study investigates the potential benefit of additional auditory stimulation (with temporal regularity or not) on visual sequence learning, as implemented by a classical SRTT. The study is timely and novel, proposing to combine temporal regularity processing with prediction and sequence learning. The project is very interesting and promising. However, the design with 6 between-participants conditions and only 10 participants per condition is ambitious and might lead to underestimate the effects or the missing observation of differences for RTs. The manuscript is missing numerous details and requires some explanations and clarifications (see below). Some streamlining of the writing and section presentation should also help the reader.

The manuscript could also benefit from integrating the present work into other previous research. Numerous statistical learning studies investigating the influence of concurrent or implemented temporal structures are missing in the introduction and discussion (e.g., Buchner & Steffens, 2001; Shin & Ivry 2002; Selchenkova 2014a,b). The paper might also benefit from the integration of the Dynamic Attending Theory and its hypothesis of metric binding (see for example Jones, 2016, 2019). Further relevant references for the present work are the papers by Fujii & Wan (2014), Patel & Iversen (2014) and Tierney & Kraus (2014).

Buchner, A., and Steffens, M. C. (2001). Simultaneous learning of different regularities in sequence learning tasks: limits and characteristics. Psy- chol. Res. 65, 71–80.
Fujii, S., & Wan, C. Y. (2014). The role of rhythm in speech and language rehabilitation: The SEP hypothesis. Frontiers in Human Neuroscience, 8.
Jones, M. R. (2016). Musical time. In S. Hallam, I. Cross, & M. Thaut, The Oxford Handbook of Music Psychology (2nd ed.). Oxford University Press.
Jones, M. R. (2019). Time will tell. Oxford University Press.
Patel, A. D., & Iversen, J. R. (2014). The evolutionary neuroscience of musical beat perception: The Action Simulation for Auditory Prediction (ASAP) hypothesis. Frontiers in Systems Neuroscience, 8.
Selchenkova et al (2014a). Metrical presentation boosts implicit learning of pitch structures. PLOS ONE 9(11): e112233
Selchenkova et al. (2014b). The influence of temporal regularities on the implicit learning of pitch structures. Quarterly Journal of Experimental Psychology, 67, 2360–2380.
Shin, J. C., & Ivry, R. B. (2002). Con- current learning of temporal and spatial sequences. J. Exp. Psychol. Learn. Mem. Cogn. 28, 445–457.
Tierney, A., & Kraus, N. (2014). Auditory-motor entrainment and phonological skills: Precise auditory timing hypothesis (PATH). Frontiers in Human Neuroscience, 8.

- Thanks for these recommendations. We read these articles and integrated some of these previous researched into the present work.

Introduction (p.3): “More precisely, Shin and Ivry (32) showed that temporal patterns can be learned when the intervals are associated with concrete events, such as specific visual stimuli or finger movements, and that temporal and spatial parameters are learnt in an integrated fashion allowing to acquire the order of a repeated sequence.”

Discussion (p.21-22): Interestingly we found facilitation in the RhythAud condition even if we did not manipulate directly the temporal pattern of the to-be-learned material as it was done in most previous studies (see for example 32, 89, 90). Indeed, the sequence of position was played out through the visual modality whereas we implemented a temporal structure through auditory stimuli. One hypothesis is that the temporal regularity of these stimuli could have prepared the attentional system to deal with specific stimuli arriving in the same temporal pattern (e.g., 91,92). Indeed, these effects have already been shown using other tasks of implicit learning of pitch structures (94,95), working memory (96), and statistical learning of artificial language (97).”

- 89. Willingham, D. B., Greenberg, A. R., & Thomas, R. C. (1997). Response-to-stimulus interval does not affect implicit motor sequence learning, but does affect performance. *Memory & Cognition*, 25(4), 534-542.
- 90. Rünger, D. (2012). How sequence learning creates explicit knowledge: the role of response–stimulus interval. *Psychological research*, 76(5), 579-590.
- 91. Jones, M. R., & Boltz, M. (1989). Dynamic attending and responses to time. *Psychological review*, 96(3), 459.
- 92. Large, E. W., & Jones, M. R. (1999). The dynamics of attending: How people track time-varying events. *Psychological review*, 106(1), 119.
- 94. Selchenkova et al (2014a). Metrical presentation boosts implicit learning of pitch structures. PLOS ONE 9(11): e112233
- 95. Selchenkova et al. (2014b). The influence of temporal regularities on the implicit learning of pitch structures. Quarterly Journal of Experimental Psychology, 67, 2360–2380.
- 96. Plancher, G., Lévêque, Y., Fanuel, L., Piquandet, G., & Tillmann, B. (2018). Boosting maintenance in working memory with temporal regularities. *Journal of Experimental Psychology: Learning, Memory, and Cognition*, *44*(5), 812.
- 97. Hoch, L., Tyler, M. D., & Tillmann, B. (2013). Regularity of unit length boosts statistical learning in verbal and nonverbal artificial languages. *Psychonomic bulletin & review*, 20(1), 142-147.

Methods
- For the musical background of the participants, it would be relevant to further add the information of musical training in terms of average and SD. Table 1 does not present the participant characteristics (see page 5). One table seems to be missing and the reference to table 1 related to the pre-test condition should read table 2?

- Thank you for this comment, we agree that it would have been interesting to know more about participants’ musical background to see if this influences the auditory stimuli effects. However, we just ensured that they did not practice or practiced music regularly (more than two hours per week for a maximum of two years) and we did not ask them more precisely their background.

We removed the sentence « Characteristics of participants are summarized in Table 1 » to match the Table references. Table 2 has been completed with phasic alert and divided attention scores.

- Response-Stimulus-Interval was 200ms. In absence of response, the highlighted square remained on the screen for 3000ms. Did the sequence then go on with the next highlighted square? Please clarify.

- Thanks for this clarification. The sentence was completed to be more clear: “If the participant did not press any key, the stimulation remained for 3000ms on the screen and then went on with the next stimulus » (p.7).

- Please clarify the construction of B6. Does “same pseudo-random sequence following the same rules” now refer to all 100 stimuli? Or also a repeating sequence of 10 cycling through ten times?

- Thanks, we agree that the previous sentence was not clear enough. We clarified it: “participants performed the same pseudo-random sequence following the previous rules applied to 100 trials.” (p.8).

- Considering the discussions in SRTT research about what is learned during exposure, why didn’t the authors use two controlled Second-Order-Conditional sequences (see research by Cleeremans or Destrebecqz), counterbalancing as test or training across participants?

- It is an interesting comment. Indeed, it is possible that the transfer from the learned sequence and the random presentation is not equal for all sequences used in the general learning phase. However, given that all the four different sequences were equally distributed across conditions, the effect should be equal in all conditions. Moreover, Second-Order-Conditional sequences are specifically designed to investigate implicit learning by reducing the easiness to detect the regularities. Given that implicit and explicit processes were not our main research interest, we have not used such a design. We did not change the manuscript but we can if required.

- Why was the first block referred to as B0? What was the difference between B0 and B1? I guess B0 was presented without auditory stimulation, please clarify.

- Thanks for this question. We agree that the difference between B0 and B1 was not clear. B0 was another sequence than B1 (and thus different from B2, B3, B4 and B5). Its purpose was to check if there were some differences between groups prior to the experiment (like a baseline).

B0 was performed in the same condition as the other blocks (see previous changes). Introducing auditory stimulations in B1 in some conditions but not all might have induced unpredictable effects on participants’ performance in these conditions and thus complicated the interpretation of the general learning phase.

We have added clarification about B0 in the manuscript: “To make sure that there were no significant differences between the groups in performance at the beginning of the experiment, a first Block (called B0) was presented prior to the experiment phase (as a baseline). This Block consisted of a sequence of 10-items repeated ten times (100 trials). This sequence was randomly picked out of the four following sequences and was performed in the same condition as the following blocks.” (p.9) and “Each sequence was attributed in a counterbalanced manner to the participants for each condition. The sequence practiced in the following blocks was different from the one selected for B0” (p.9).

- Page 7 “This sequence was randomly assigned to each participant” – Does this refer to one of the four sequences (A, B, C or D)? Why were these sequences not respectively used as the test-block B6 sequences (such as, for example, Participant 1 would get sequence A for blocks B0 to B5 and sequence B for block 6 while Participant 2 would get the reverse attribution)?

- Thanks for this comment. The main goal of our experiment was to compare the sequence learning between several conditions and not the transfer effect. Using reverse attributions decrease unpredictability in the sixth block and leave room for learning the novel sequence what might hinder partially the interference effect. Indeed, as shown by Du et al., (2016), sequence learning occurs both within and between blocks, even in the “random” block when a repeated sequence is presented. We did not add to the manuscript but we can if required.
- Du, Y., Prashad, S., Schoenbrun, I., & Clark, J. E. (2016). Probabilistic motor sequence yields greater offline and less online learning than fixed sequence. *Frontiers in Human Neuroscience*, 10, 87.

- Why did the authors not include the expected B7 block that returns back to the exposure sequence (B0 to B5), as usually done in SRTT?

- We totally agree that it would have been interesting to include B7 with a return back to the exposure sequence. This block would have helped us to figure out if auditory stimulations also facilitate the reactivation of the sequence. Some articles do not include this block (e.g., Bo et al., 2011; Destrebecqz, 2004; Robertson, 2007) but this is explicitly proposed as perspective in the conclusion part “these preliminary results need for replications and extension with a retention test (with reintroduction of the repeated sequence in another Block, B7)” (p.22)
- Bo, J., Jennett, S., & Seidler, R. D. (2011). Working memory capacity correlates with implicit serial reaction time task performance. *Experimental brain research*, 214(1), 73.
- Destrebecqz, A. (2004). The effect of explicit knowledge on sequence learning: A graded account. *Psychologica Belgica*, 44(4).
- Robertson, E. M. (2007). The serial reaction time task: implicit motor skill learning?. *Journal of Neuroscience*, 27(38), 10073-10075.

- The material presentation requires further details: what was the irregular pattern used in IAS? Was it the same for all blocks? If yes, participants might have learned it and it might have become less unexpected over time.

- Thanks for this comment. The irregular pattern was composed of the same number of auditory stimulations as the regular one but with irregular intervals. The same pattern was presented across all blocks of the IAS condition. This pattern was programmed with the free software Audacity in a pseudo-random fashion to produce random auditory stimulations. Given its complexity it is very unlikely that participants learned it. Literature shows that it is extremely difficult to synchronize with irregular pattern of stimuli (e.g., Repp & Su, 2013). Although it is possible to learn an irregular pattern of 11 stimuli with repeated practice (e.g., Lê et al., 2021; Blais et al., 2021), the pattern proposed in the present experiment correspond to 160 stimuli. Thus it is unlikely that participants were able to learn such a long pattern. Even if they did, our results show that irregular pattern does not help participants to learn the sequence.

The text has been completed as follow: “There was the same number of auditory stimulations in this soundtrack than in the RhythAud condition but intervals between them were determined randomly. This pattern was programmed with the free software Audacity in a pseudo-random fashion to produce random auditory stimulations. The same soundtrack was presented in all blocks.” (p.9).

- Repp, B. H., & Su, Y. H. (2013). Sensorimotor synchronization: a review of recent research (2006–2012). *Psychonomic bulletin & review*, 20(3), 403-452.
- Blais, M., Jucla, M., Maziero, S., Albaret, J. M., Chaix, Y., & Tallet, J. (2021). The Differential Effects of Auditory and Visual Stimuli on Learning, Retention and Reactivation of a Perceptual-Motor Temporal Sequence in Children With Developmental Coordination Disorder. *Frontiers in Human Neuroscience*, 15, 156.
- Lê, M., Blais, M., Jucla, M., Chauveau, N., Maziero, S., Biotteau, M., ... & Tallet, J. (2021). Procedural learning and retention of audio‐verbal temporal sequence is altered in children with developmental coordination disorder but cortical thickness matters. *Developmental science*, 24(1), e13009.

- Was the auditory material presented during all blocks (B0 to B6)?

- Yes, we clarified that B0 was performed in the same condition as the other blocks in order to not induce transfer of learning or adaptations at the beginning of practice of the sequences (see previous response and changes page 9).

How was the sound of the auditory stimulation made? At which pitch height and loudness level was it presented? How was it presented (via headphones or free field)?

- The sound was presented via external computer speakers in an isolated and quite room. The text was completed as follow page 9: “In CongrAV, NonCongrAV, RhythAud, IrregAud and FastRhyth conditions the auditory stimulations were presented via external speakers and consisted in a 500Hz, 100ms sinewave presented at 80dB.”

- What was told to the participants about the purpose of the sound?

- “Participants were told that there would be auditory stimuli but we did not tell them anything about their purpose”. This information is added page 9 (methods section).

- When were the TAP tests presented?

- TAP and SRTT were presented in a counterbalanced order between participants. It was clarified page 10: “The order of SRTT and TAP tests was counter-balanced for each participant to prevent training or fatigue effects.”

- For the explicit knowledge testing, why the choice or 3-alternatives rather than a subjective scale with more gradings, which might be more sensitive to differences?

- We agree that a subjective scale with more grading, would have been more sensitive to differences in explicit learning of the sequence. It is an interesting question to investigate if auditory stimulations influence implicit and explicit processes in the task. To determine whether performance is due to implicit or explicit processes several techniques can be used such as inclusive and exclusive production (Destrebecqz & Cleeremans, 2001; Stefaniak, et al., 2008; Wilkinson & Shanks, 2004) or sequence recognition tasks (Perruchet & Amorim, 1992; Willingham et al., 1993; Sanchez-Mora & Tamayo, 2021). Even if it was not our main research question, we have added that “Further investigations about explicit knowledge associated to learning with and without auditory stimuli are needed, for example with subjective scale with more gradings, which might be more sensitive to differences” (page 17-18, discussion).
- Destrebecqz, A., & Cleeremans, A. (2001). Can sequence learning be implicit? New evidence with the process dissociation procedure. *Psychonomic bulletin & review*, 8(2), 343-350.
- Stefaniak, N., Willems, S., Adam, S., & Meulemans, T. (2008). What is the impact of the explicit knowledge of sequence regularities on both deterministic and probabilistic serial reaction time task performance?. *Memory & Cognition*, 36(7), 1283-1298. Wilkinson, L., & Shanks, D. R. (2004). Intentional control and implicit sequence learning. *Journal of Experimental Psychology: Learning, Memory, and Cognition*, 30(2), 354.
- Perruchet, P., & Amorim, M. A. (1992). Conscious knowledge and changes in performance in sequence learning: Evidence against dissociation. *Journal of Experimental Psychology: Learning, Memory, and Cognition*, 18, 785–800.
- Willingham, D. B., Greeley, T., & Bardone, A. M. (1993). Dissociation in a serial response time task using a recognition measure: Comment on Perruchet and Amorim (1992).
- Sánchez-Mora, J., & Tamayo, R. M. (2021). From incidental learning to explicit memory: The role of sleep after exposure to a serial reaction time task. *Acta Psychologica*, 217, 103325.

Results:
- Were RTs only kept from correct responses? It is not clear how the average and the variability was calculated: “across Blocks of trials were computed” Wouldn’t it need to be done across trials of each of the blocks?

- Thanks for this correction, the previous formulation was ambiguous. We modified the text as proposed “Average reaction times (RTmean), variability of reaction times (RTsd) and errors for all participants in each Condition were computed across trials of each block” (page10).

We also added the following sentence to clear up this point: “For all analyses, incorrect responses were not included in the RT analyses.” after the title “Data analysis” (p.11). Given the high correct response rate, it is reasonable to assume that when an error occurred, the process being studied was not carried out "normally”. Thus, the interpretation of correct and incorrect RTs together might be difficult. Moreover, most of SRTT studies also remove incorrect responses in the RT analysis (e.g., Thomas & Nelson, 2001; Smith et al., 2001; Curran, 1997; Nissen & Bullemer, 1987; Willingham et al., 1989)

- Thomas, K. M., & Nelson, C. A. (2001). Serial reaction time learning in preschool-and school-age children. *Journal of experimental child psychology*, *79*(4), 364-387.
- Smith, J., Siegert, R. J., McDowall, J., & Abernethy, D. (2001). Preserved implicit learning on both the serial reaction time task and artificial grammar in patients with Parkinson's disease. *Brain and Cognition*, 45(3), 378-391.
- Curran, T. (1997). Higher-order associative learning in amnesia: Evidence from the serial reaction time task. *Journal of cognitive neuroscience*, 9(4), 522-533.
- Nissen, M. J., & Bullemer, P. (1987). Attentional requirements of learning: Evidence from performance measures. *Cognitive psychology*, 19(1), 1-32.
- Willingham, D. B., Nissen, M. J., & Bullemer, P. (1989). On the development of procedural knowledge. *Journal of experimental psychology: learning, memory, and cognition*, 15(6), 1047.
- Figure 2 could integrate an extra data point presenting RTs at B0 (without connecting lines to B1).
- Thank you for this proposition. As recommended, we added B0 in all the Figures.

- Should title 2.6. read “data analyses”? This information could be combined with the data analysis section further up, allowing for streamlining the manuscript and removing redundancies.

- We changed subtitles names and removed the third level title “data analysis”.

- 2.6.1: The correlations should also be completed with the task learning score B0-B5 for attention and B0 analyses.

- The correlations were computed; they are not significant. The information has been added page 12: “Pearson’s correlations were used to explore the link between attentional performance (alert phasic and divided attention) and the SRTT general learning score (difference B1-B5 = RTmeanB1-B5, RTsdB1-B5, ErrorB6-B5) and specific learning score (difference B6-B5 = RTmeanB6-B5, RTsdB6-B5, ErrorB6-B5)”; “Pearson’s correlations were used to explore the link between B0 performance (RTmean, RTds and errors) and the SRTT general learning score (difference B1-B5 = RTmeanB1-B5, RTsdB1-B5, ErrorB6-B5) and specific learning score (difference B6-B5 = RTmeanB6-B5, RTsdB6-B5, ErrorB6-B5).” (p.12)

- 2.6.2: the two ANOVAs are redundant – using blocks as additional factor (to investigate the difference between B5 and B6) or running the analysis on the computed difference should provide equivalent results (as shown by same F-values, p-values and partial eta2, see page 13).

- We agree. Thank you for this comment, the first ANOVA was removed.

- Page 11: Please present the used categories of the Bayes factor interpretation in the text.

- Here is the table we used for the interpretation of each Bayes factors level. It is adapted from Jeffreys (1998). We have not added this table in the manuscript but we can add it in the appendix section if required.

| BF10 | Support for hypothesis |
| --- | --- |
| < 0.01 | Decisive evidence for H_0_ |
| 0.03-0.01 | Very strong evidence for H_0_ |
| 0.10-0.03 | Strong evidence for H_0_ |
| 0..33-0.10 | Substantial evidence for H_0_ |
| 0.33-1 | Anecdotal evidence for H_0_ |
| 1 | No evidence |
| 1-3 | Anecdotal evidence for H_1_ |
| 3-10 | Substantial evidence for H_1_ |
| 10-30 | Strong evidence for H_1_ |
| 30-100 | Very strong evidence for H_1_ |
| >100 | Decisive evidence for H_1_ |

- 3. Results:
o Page 12: add exact p-values for the correlations into the text (or the table). Were Bayesian analyses also run for these pre-test analyses (as for 3.2)?

- Table 2b have been added with all results. Table 2a with pre-tests results have been completed with Phasic Alert and Divided attention scores (and comparisons between groups). Given that these analyses were secondary, we have decided to not carry out Bayesian’s statistic but we can do if required.

o Page 13: why were additional Friedman tests ran for IAS?

- All conditions were tested but we only reported significant results. We completed the sentence. We clarified it in the text “For general learning, Friedman tests revealed a significant Block effect only in the IrregAud Condition for both errors (X²R (10, 4) = 11.55, p = .021) and RTsd (X²R (10, 4) = 10.96, p = .027)” (p.16)

Figure 2 suggests that the RTs differ in absolute terms between at least a subset of the conditions (e.g., fastest RT for RAS, followed by VO, QRAS and IAS being slowest). It might be interesting to normalize response times (e.g., using z-scores for each participant). This would remove these inter-group differences in terms of absolute response speed, but might allow for being more sensitive to reveal differences in general and specific learning.

- We agree with Reviewer 2 that a z-score might be more sensitive to general and specific learning. However, the systematic review of Zwartz et al. (2019) reported that normalized data might be difficult to interpret and support the use of raw RT data rather than normalized RT data even when baseline speed differences are present. Moreover, they precise that studies who examined normalized data as well as raw RT data found similar results (Barnes et al., 2008; Brown et al., 2010; Gabriel et al., 2015).

That is why we computed ANOVA of z-scores standardized by participants, but we did not include it in the manuscript. We can add it if required. Each person’s observation on each measure was converted to a z score standardized on participant’s mean and variance (pooled across blocks). As previous, ANOVA revealed significant block effect on both general learning (*F* (4,236) = 23.67, *p* < .001, η^2^_P_ = .286, BF_10_ > 1000) and specific learning (*F* (1, 59) = 393.21, *p* < .001, η^2^_P_ = .870, BF_10_ > 1000).

- Zwart, F. S., Vissers, C. T. W., Kessels, R. P., & Maes, J. H. (2019). Procedural learning across the lifespan: A systematic review with implications for atypical development. *Journal of Neuropsychology*, *13*(2), 149-182.

Discussion
- Page 18/19: The discussion of the QRAS condition is interesting, but it requires that the overall speed of response of the participants in this condition is not faster (as faster RTs would leave less room for improvement, that is decrease of RTs)

- We agree to this remarks, so we checked more accurately the difference in RT between conditions. Using the same procedure as for the previous comment, we computed an ANOVA with z-scores standardized by block. That is, each person’s observation on each measure was converted to a z score standardized on the block mean and variance (pooled across conditions). The repeated measure ANOVAs did not reveal significant effect in general learning phase (from B1 to B5) (*F* (5, 54) = 2.01, *p* = .093, η^2^_P_ = .156, BF_10_ = 2.39), or in specific learning phase (from B5 to B6) (*F* (5, 54) = 1.73, *p* = .143, η^2^_P_ = .138, BF_10_ = 0.73). Thus, it confirms the ANOVA presented in the manuscript, there is no differences in RT between conditions. We did not change the manuscript but we can if required.

- Page 19: This study showed some learning on errors but not on RTs. Was RTs particularly fast in comparison to other studies? The explanation that error modulation with RAS reflects enhanced spatial encoding does not seem to be complete because one could also argue that enhanced spatial encoding of the motor sequence should lead to modulation of RTs.

- It is an interesting remark. Actually, there is both learning on errors and on RT (both ANOVAs revealed block effects) but it is true that the differences in learning between conditions only appears on errors. We completed the discussion section (p. 18-19) with the following sentence: “Interestingly, Vékony et al. (2020) showed that performance of accuracy and speed depend on the instructions (more directed to speed or accuracy) but the learning effect occurs in both cases. Given that our results show differences in conditions on errors only, it is possible that our instructions were more emphasized on accuracy than speed.”

We also included our exact instructions in the method section (p.7). “Try to go as fast as possible and make as less as possible mistakes”.

- 77. Vékony, T., Marossy, H., Must, A., Vécsei, L., Janacsek, K., & Nemeth, D. (2020). Speed or accuracy instructions during skill learning do not affect the acquired knowledge. Cerebral Cortex Communications, 1(1).

The manuscript could gain in clarity and should be checked by a native English speaker too. At some points, clarity in writing could be increased (e.g., “Attentional performance was assessed to explore the link between visuo-motor learning and attentional skills. They were also used to make sure that groups …”) I guess ‘they’ refers to the two attentional tests, but they were not mentioned here.

- Thanks for this suggestion, we replaced “They were also used to make sure that groups…” “This measure was also done to make sure that groups did not differ in terms of attentional functions” (p. 7).

The abbreviations used for the six conditions are somewhat abstract and difficult to remember; replacing them by more informative ones would facilitate reading (e.g., VisOnly, CongrAV, NonCongrAV, RythAud, IrregAud, FastRyth). Also, “rhythmic” does not seem to be the best wording for the “rhythmic auditory stimulation”, in particular with its contrast to the Irregular auditory stimulation, the label “Regular auditory stimulation” (RegAud) seems more appropriate.

- We acknowledge reviewer’s comment and modify the manuscript accordingly. “VO”, “CAV”, “NCAV”, “RAS”, “IAS”, “QRAS” and “rhythmic auditory stimulation” are replaced by “VisOnly”, “CongrAV”, “NonCongrAV”, “RythAud”, IrregAud, FastRyth” and “Regular auditory stimulation” (RegAud). Modifications are made to the title, abstract and where appropriate throughout the manuscript in particular in the Material-Methods, Results, and Discussion sections.
