# Supplementary material for: Regular rhythmic and audio-visual stimulations enhance procedural learning of a perceptual-motor sequence in healthy adults: A pilot study
Source: PLoS One. 2021 Nov 15;16(11):e0259081. doi: 10.1371/journal.pone.0259081 (PMC8592429; doi:10.1371/journal.pone.0259081)
Supplement: Attachment — Submitted filename: Responses to Reviewers.docx [file pone.0259081.s006.docx]

Dear Professor Fetsch and reviewers,

We would like to thank you for your positive and constructive comments on our manuscript entitled “*Regular rhythmic and audio-visual stimulations enhance procedural learning of a perceptual-motor sequence in healthy adults: a pilot study*”.

The manuscript has been revised for better readability according to the suggestions. We responded to all of the suggestions with associated changes highlighted in blue.

All authors have reviewed and agreed to the submission of the revised manuscript. We hope that our responses fix all the concerns that you raised and that the manuscript is now acceptable for publication. Please do not hesitate to contact me if there are any questions.

**Editor:** Thank you for submitting your manuscript to PLOS ONE. After careful consideration, we feel that it largely meets PLOS ONE’s publication criteria, although there are a small number of remaining issues to address and suggestions for improvement. Therefore, we invite you to submit a revised version of the manuscript that addresses these issues.

Both reviewers and I appreciate the effort in response to the previous comments, and all agree the resulting manuscript is greatly improved. Reviewer 2 listed a number of relatively minor concerns/suggestions, and one or two more substantial ones, that should be addressed.

While revising your submission, please upload your figure files to the Preflight Analysis and Conversion Engine (PACE) digital diagnostic tool, https://pacev2.apexcovantage.com/. PACE helps ensure that figures meet PLOS requirements. To use PACE, you must first register as a user. Registration is free. Then, login and navigate to the UPLOAD tab, where you will find detailed instructions on how to use the tool. If you encounter any issues or have any questions when using PACE, please email PLOS at figures@plos.org. Please note that Supporting Information files do not need this step.

- Thank you for this recommendation. We uploaded all our figure files to the PACE tool and all of them met PLOS requirements.

**Reviewer #1:** I’d like to thank the authors for their response. I believe the inclusion of Figure 4a, and block b0 in multiple figures, paint a more clear picture of what’s going on.

Moreover, thank you for the general vs. specific learning clarification. I originally understood general learning as “total” learning, i.e. something that would encompass specific learning; here however general and specific learning are distinct.

1) My one remaining suggestion is that you add a short definition earlier in the manuscript, when these terms first appear, as not all readers might be familiar with the distinction.

- We acknowledge reviewer’s comment and included a definition of these terms in the introduction section: “Indeed, as opposed to the general learning that includes both familiarization and sequence learning, this difference in RT (or errors) reflects the learning of the specific sequence and is called specific learning” (p.2).

**Reviewer #2:** The revised manuscript is considerably improved in clarity and presentation. I still have the following points that the authors should address in a revision.

- Thank you for your positive appreciation.
- The manuscript should address the concern of the ambitious design of six between-participants conditions and only 10 participants per condition, which might lead to underestimate the effects, for example.
- We agree that the ambitious design and the small sample might have led to underestimate the effects. This information has been added in the manuscript p.24: “Note that we used a very ambitious design with six different conditions. However, all of them were required to understand the overall effect. For example, we showed that it is not only the regularity of the tempo that is decisive but that this tempo is in adequacy with the motor task to facilitate the learning. Moreover, the small sample might have led to underestimate the effects. However, a within-participants design would not have been possible because of (1) the possible transfer or interference effects between conditions and (2) the length of the experiment (6 different conditions x 6 blocks of 100 stimuli + attentional tests). Despite this ambitious design, we found some promising results that need to be explored more deeply and replicated.”

- The response letter explains that B0 was another sequence different from the to-be-learned sequence and supposed to act as baseline. However, this would require to be presented without auditory stimulation. If B0 is presented with the same auditory condition as B1, then its purpose is not clear. Indeed, then even B0 is submitted to potential influences of the experimental condition. Using the first block to check that participants did not differ between groups would have served the same purpose. Considering that within-block learning can occur, presenting two different sequences at the beginning might also affect learning (i.e., learning and re-learning) and raises concerns regarding the similarity between B0 and the experimental sequence, which might affect learning differently.
- We agree and explain our decision in the manuscript (p.10): “Note that B0 is performed in the same condition as the following Blocks because changing the way how stimuli are introduced between B0 and B1 would have possibly induced confounding effects on participants’ performance, hence confusing the interpretation of the results of the general learning phase”
- Page 84 (pdf) “following the previous rules applied to 100 trials” (p. 8) This “citation” is not in the manuscript, please check all citations to make sure that the manuscript contains the same text as claimed in the response letter to the reviewers.
- Thank you for this remark. We apologize for this mistake. The sentence was completed in the manuscript (p.8).
- Page 84 (pdf) “applied to 100 trials” “trials” is a terminology that might lead to confusion, “items” seems more appropriate (here and elsewhere) or clarify. Keep the same wording throughout (sometimes referred to as “stimulations”).
- Thanks for this comment. We replaced “stimulations” and “trials” by “items” when appropriate throughout the manuscript.
- The suggestion to use the same sequences across participants as test or learning was not to investigate “transfer effects” (page 85 of the pdf), but to control for sequence specific features. Please clarify and integrate your explanation and justification also in the manuscript as other readers might wonder the same.
- We agree that this issue needs clarifications. “We used several different sequences instead of a unique one because learning can depend on the sequence used (64) and it opens up the possibility that the obtained results may be specific to a particular fixed sequence. Thus, each group was we proposed different controlled sequences sharing the same rules.” (pages 8).

- 64. DeCoster, J., & O’Mally, J. (2011). Specific sequence effects in the serial reaction time task. *Journal of motor behavior*, *43*(3), 263-273.
- Page 53 pdf/page8 manuscript: “a last Block (B6) presented the visual stimulations in a pseudo-random fashion”. The authors should spell out the exact sequence used (as they do for sequences A, B, C and D) so that the reader can compare it with the different training sequences used.
- Thanks for this remark, we added the random sequence in the manuscript “3,2,1,3,4,1,3,4,2,3,1,2,4,3,1,2,3,4,2,3,1,4,3,2,4,1,2,4,1,3,2,4,1,2,4,1,3, 4,2,1,4,2,3,1,4,3,1,2,4,3,1,2,4,3,1,2,4,3,1,2,4,2,3,1,4,1,3,2,1,3,4,2,1,3,2,1,3,2,1,4,2,3,1,4,3,2,4,1,2,4,1,3,4,2,3,4,1,4,2,3” (p.10)
- Page 86 pdf: The temporal pattern consisted of 160 stimuli. This needs to be clarified as one block consisted of 100 items. In addition, the authors should explain how the “intervals between them were determined randomly”, what were the possible intervals (range min/max, sampling etc). Also provide the used sequence as supplementary material in written form and audio file.
- The mean time required to perform a block by participants in the pilot data was around 60 seconds [(RT + RSI) * Nb stimuli = (350ms + 250ms) * 100 = 60000ms = 60s). Thus, we created a soundtrack of 75s so that slower participants could have auditory stimulations until they finish. Given that auditory stimulations were presented every 500ms, and that the auditory stimulation lasts 100ms, the soundtrack was composed of 125 auditory stimulations. This number is not linked with the number of visual stimuli. The “intervals were pseudo-randomly generated in a range of 0.022s to 2.891s and a mean of 0.494s.” (p.10).
- It would be helpful for the readers to appreciate the work done by the authors and the manipulations used by adding as supplementary materials some short video excerpts displaying the paradigm and illustrating the auditory congruency or not (e.g., allowing for evaluating also the potential disturbance of the irregular sequence).
- We agree that it could be a good idea to clarify the materials so we added the two soundtracks in supplementary materials.
- page 87. The presentation at 80dB seems quite loud. Why did the authors opt for this loudness level?
- We opted for this level to be sure that participants could not ignore the auditory stimuli. We completed the information regarding the way how sounds were presented in the manuscript (P.11): “In CongrAV, NonCongrAV, RegAud, IrregAud and FastRhyth conditions the auditory stimuli were presented via two external speakers placed on both sides of the screen and consisted in a 500Hz, 100ms sinewave presented at 80dB.”
- Page 89 (pdf): I agree with the authors that Jeffreys table could be placed in an appendix section.
- We added Jeffreys table in the supplementary materials.
- Page 90: Thanks for this response. The authors should present their normalized RT data also into the manuscript, as they propose here. The z-score transformation (pooled across blocks) should remove the between-group speed differences and allow for the discussion (previous version page 18/19 of the manuscript). However, I do not see the purpose to use z-scores standardized by block as this does not allow for testing learning effects (see bottom part of page 90).
- We included the normalized data also in the manuscript as Figure 2b (Figure 2 was renamed Figure 2a).

We also included the following sentences in the manuscript:

- p. 16. “In order to remove inter-group differences in terms of absolute response speed and allow for more sensitive differences in general learning, each participant’s observation on each measure was converted to a z score standardized on participant’s mean and variance (pooled across blocks). As previous, ANOVA revealed significant block effect on general learning phase (*F* (4,236) = 23.67, *p* < .001, η^2^_P_ = .286, BF_10_ > 1000) (Fig 2b).”
- p. 17. “In order to remove inter-group differences in terms of absolute response speed and allow for more sensitive differences in specific learning, each participant’s observation on each measure was converted to a z score standardized on participant’s mean and variance (pooled across blocks). As previous, ANOVA revealed significant block effect on specific learning phase (*F* (1, 59) = 393.21, *p* < .001, η^2^_P_ = .870, BF_10_ > 1000) (Fig 2b)”.

Indeed, the z-score by block does not allow for testing learning effects but it shows more directly the differences between the groups. We agree that it is not necessary to include it in the manuscript.

- page 10 manuscript: “fixed to randomized” - pseudorandomized?
- Right, we modified accordingly in the manuscript (p.10).
- page 19 manuscript: more difficult than rhythmic auditory stimulations” - does this here refer to “regular” rhythmic stimulations? Please clarify (and check throughout the manuscript that the labelling is clear).
- Thanks for this remark. We agree that “regular rhythmic auditory stimulations” is more appropriate than “rhythmic auditory stimulations”. We replaced when appropriate throughout the manuscript including the title.
- The authors should also extend their discussion to the influence of multi-dimensional or dual cues in learning (e.g., Mitchel & Weiss, 2011 JEP:LMC).
- We agree that this literature had to be included in the discussion. We added the following paragraph “Our results also contribute to the debate in the literature distinguishing (1) a modality-specific mechanism proposing that learning occurs in each modality separately and (2) a modality-general mechanism in which learning is independent across modalities (91,94). In line with previous results (91), our results tend to be in favor of the latter purpose. Indeed, in (91) authors showed that learners are able to extract statistical regularities from audiovisual input and to integrate it into audio and visual streams separately. In our case, even if the auditory stimulations alone don’t provide any cue regarding the sequence, it seems that they still helped the learning of the visual sequence when they were presented simultaneously with the visual stimulations. Therefore, our results are in line with the purpose that procedural learning is sensitive to multimodal input.” (p.23).
  - 91. Mitchel AD, Weiss DJ. Learning across senses: Cross-modal effects in multisensory statistical learning. *Journal of Experimental Psychology: Learning, Memory, and Cognition*. 2011;37(5):1081–91.
  - 94. Seitz AR, Kim R, van Wassenhove V, Shams L. Simultaneous and Independent Acquisition of Multisensory and Unisensory Associations. *Perception*. 2007 Oct 1;36(10):1445–53.
